# Screening under infection-relevant conditions reveals chemical sensitivity in multidrug resistant invasive non-typhoidal *Salmonella* (iNTS)†

**DOI:** 10.1039/d3cb00014a

**Published:** 2023-07-08

**Authors:** Caressa N. Tsai, Marie-Ange Massicotte, Craig R. MacNair, Jordyn N. Perry, Eric D. Brown, Brian K. Coombes

**Affiliations:** a Department of Biochemistry & Biomedical Sciences, McMaster University Hamilton ON L8S 4L8 Canada coombes@mcmaster.ca; b Michael G. DeGroote Institute for Infectious Disease Research Hamilton ON Canada; c Farncombe Family Digestive Health Research Institute Hamilton ON Canada

## Abstract

Bloodstream infections caused by invasive, non-typhoidal *Salmonella* (iNTS) are a major global health concern, particularly in Africa where the pathogenic variant of *Salmonella* Typhimurium sequence type (ST) 313 is dominant. Unlike *S.* Typhimurium strains that cause gastroenteritis, iNTS strains cause bloodstream infections and are resistant to multiple first-line antibiotics, thus limiting current treatment options. Here, we developed and implemented multiple small molecule screens under physiological, infection-relevant conditions to reveal chemical sensitivities in ST313 and to identify host-directed therapeutics as entry points to drug discovery to combat the clinical burden of iNTS. Screening ST313 iNTS under host-mimicking growth conditions identified 92 compounds with antimicrobial activity despite inherent multidrug resistance. We characterized the antimicrobial activity of the nucleoside analog 3′-azido-3′-deoxythymidine as an exemplary compound from this screen, which depended on bacterial thymidine kinase activity for antimicrobial activity. In a companion macrophage-based screening platform designed to enrich for host-directed therapeutics, we identified three compounds (amodiaquine, berbamine, and indatraline) as actives that required the presence of host cells for antibacterial activity. These three compounds had antimicrobial activity only in the presence of host cells that significantly inhibited intracellular ST313 iNTS replication in macrophages. This work provides evidence that despite high invasiveness and multidrug resistance, ST313 iNTS remains susceptible to unconventional drug discovery approaches.

## Abstract 2

Invasive non-typhoidal *Salmonella* (iNTS) are a growing bacterial threat due to their antimicrobial resistance. This work demonstrates that iNTS are susceptible to host-directed therapies that inhibit intracellular bacterial replication.

## Graphical abstract

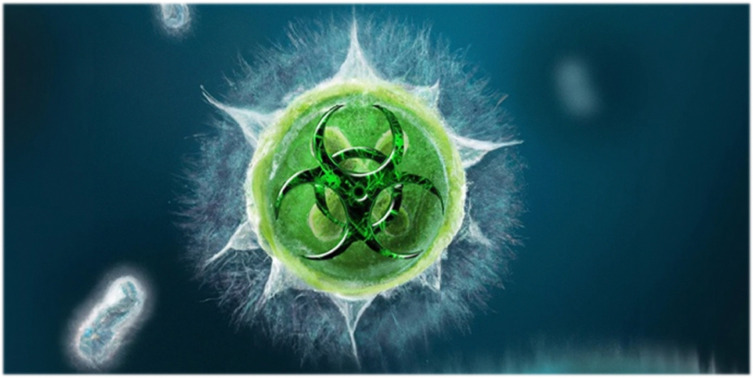

## Introduction

*Salmonella enterica* is an important global pathogen that causes disease in a wide range of animal hosts. The pathogenic potential within this species exists along a spectrum, from non-typhoidal serovars that occupy a broad host range and generally cause uncomplicated gastroenteritis, to typhoidal serovars that are host-restricted and linked to bloodstream infection.^1^ In humans, *Salmonella enterica* serovar Typhimurium commonly causes self-limiting gastroenteritis. Many cases are caused by the ST19 sequence type of non-typhoidal *S*. Typhimurium that proliferates intracellularly in epithelial cells and macrophages within *Salmonella*-containing vacuoles (SCVs). SCVs comprise a vacuolar niche for *Salmonella* that has a unique chemical composition such as low pH, limiting concentrations of divalent metals and phosphate, and is exposed to chemical cues originating from innate host defence pathways.^2,3^

New clades of non-typhoidal, highly invasive *Salmonella* have emerged that have infection courses that more closely resemble typhoidal serovars during human infection.^4^ These invasive variants cause bloodstream infections, clinically termed invasive non-typhoidal *Salmonella* (iNTS) disease.^5^ iNTS has been most studied in Africa where a single sequence type, ST313, is dominant.^6^ Most ST313 isolates are multidrug resistant to chloramphenicol, ampicillin, kanamycin, streptomycin, sulfonamides, and trimethoprim, rendering first-line treatment options ineffective and resulting in high case fatality rates.^7^ Some strains of ST313 iNTS are also resistant to third-generation cephalosporins^8^ and azithromycin.^9^

The dissemination of antibiotic resistance within iNTS is adding urgency to an already serious problem, as further restriction in treatment options for iNTS disease is likely to increase global morbidity and mortality. Given pre-existing resistance mechanisms, addressing this unmet need requires exploration of alternative therapeutics and screening platforms to identify compounds with activity against ST313 iNTS in the host setting. Several genomic, phylogenetic, and transcriptomic studies have revealed considerable genomic synteny and collinearity between ST313 and ST19 isolates.^10^ Comparisons between the two sequence types reveals greater genomic degradation in ST313, particularly in metabolic pathways required for growth in the inflamed gut, consistent with adaptation to extraintestinal pathogenic lifestyles.^10–12^ Biological work in ST313 iNTS isolates has revealed a high propensity for serum resistance,^13^ acid tolerance,^14^ colonization of the reticuloendothelial system,^15^ and decreased flagellar-based motility,^16^ stationary-phase catalase production,^17^ biofilm formation,^18^ epithelial cell invasiveness,^19^ and induction of macrophage cytotoxicity.^17^ The impact of these phenotypes on anti-infective activity against ST313 remains unknown, and a systematic study investigating the chemical sensitivity of ST313 in unconventional, infection-relevant growth conditions has not been reported.

In this work, we profiled the chemical sensitivity of ST313 iNTS to a broad range of bioactive molecules under infection-relevant conditions. We reasoned that screening in host-mimicking conditions and in intracellular environments may reveal vulnerabilities in ST313 that could be exploited as an entry point to guide future therapeutic development against iNTS disease. To this end, we performed two complementary small molecule screens against ST313 iNTS. We first screened a library of 3840 chemical compounds against ST313 iNTS grown in media that resembles the chemical properties of the intracellular SCV, looking to identify compounds with antimicrobial activity in this defined chemical niche. We characterized one exemplar active from this screen—the nucleoside analog 3′-azido-3′-deoxythymidine (AZT)—as an inhibitor of ST313 that was dependent on bacterial thymidine kinase activity and that synergized with some conventional antibiotics. We also screened the same chemical library on macrophages using a screening design aimed to enrich for host-targeted compounds that enhance host-dependent bacterial killing. By counter-selecting the resulting screening hits against the first chemical screen, we identified three compounds (amodiaquine, berbamine, and indatraline) that had no intrinsic antimicrobial activity yet significantly restricted intracellular replication of ST313 iNTS in macrophages that had been pretreated with either of these compounds. Preliminary experiments investigated possible mechanisms underlying the niche-specific growth inhibition of intracellular ST313 iNTS in the presence of host-directed therapeutics.

## Results

### Chemical sensitivity of ST313 iNTS under infection-relevant conditions

Recent studies have illustrated the value of antimicrobial susceptibility testing in physiological, infection-relevant growth environments,^20–24^ where ionic, nutrient, and stress conditions differ from conventional bacteriologic media traditionally used in screening. We previously used acidic, low phosphate, low magnesium host-mimicking media (LPM) to identify new antimicrobial actives against ST19.^23^ LPM is known to mimic the chemical and nutrient composition of the intracellular replication vacuole occupied by *Salmonella* in host cells.^25^ Growth in this host-mimicking media potentiates antibiotics with normally poor activity against Gram-negative bacteria,^23,26^ indicating that host-mimicking conditions can reveal otherwise cryptic antimicrobial activities. Our previous results also illustrate that intracellular screening platforms can further expand—and also differentiate—the accessible target space from antimicrobial chemical screens conducted in host cell-free media.^23^ Macrophage screens, for example, can identify anti-ST19 compounds that lack growth inhibitory activity in all other non-host cell conditions, including host-mimicking media.

**Fig. 1:**
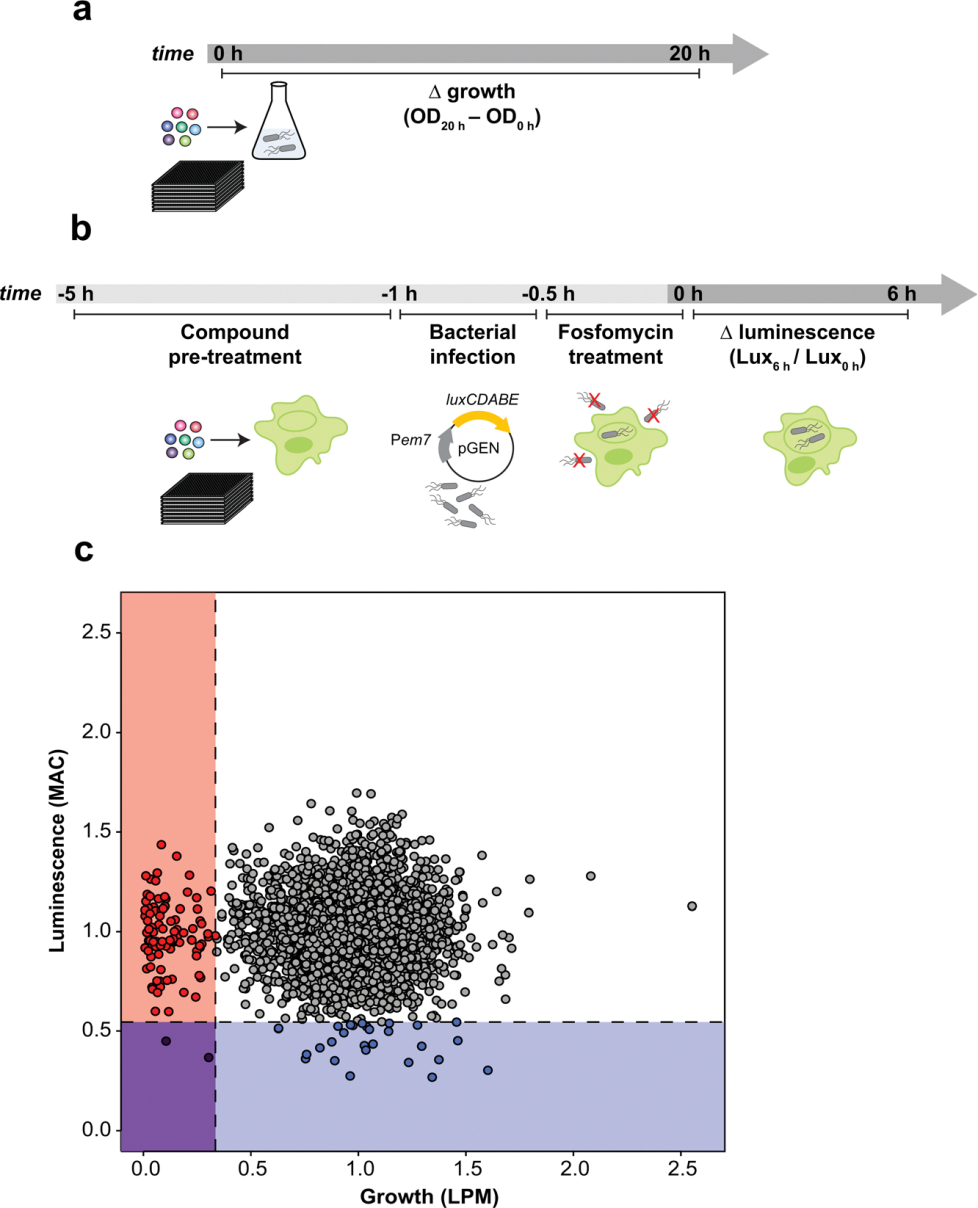
Chemical sensitivity of ST313 grown in infection-relevant conditions. (a) Workflow of screening methods for small molecule screen in LPM. OD600 was read at 0 h and 20 h after compound addition. (b) Workflow of screening methods for small molecule screen in macrophages. RAW264.7 macrophages in 384-well plates were pre-treated with compounds for 4 h, infected with ST313 expressing the luminescent pGEN-*lux*CDABE plasmid for 30 min, then treated with fosfomycin to kill extracellular bacteria for 30 min. Luminescence was read at 0 and 6 h to approximate bacterial viability. (c) Plot of screening data from macrophage and LPM chemical screens. 3840 chemical compounds were tested against ST313 iNTS grown in both conditions, each in technical duplicate. Along *x* axis, bacterial growth was monitored, values on graph represent interquartile mean-normalized, background subtracted OD600 over 20 h of incubation. Along *y* axis, luminescence production from pGEN-*lux*CDABE was monitored over 6 h, values on graph represent interquartile mean-normalized RLU per well, at 6 h divided by 0 h. Boxes and points indicate compounds that reduced bacterial growth and luminescence to 2.65 s.d. (dotted lines) below the mean of the dataset (blue, luminescence only; red, growth only; purple, both).

Seeking to maximize the accessible target space for novel iNTS therapeutics, we developed two screening approaches against ST313 that considered the systemic nature of disseminated iNTS disease and the poorly understood landscape of intracellular iNTS-immune system interactions. To this end, we screened a chemical library of 3840 previously approved drugs, natural products, and other biological actives against ST313 iNTS grown in host mimicking LPM to identify compounds with antimicrobial activity as determined by growth after 20 h of incubation (Fig. 1a). Second, we screened the same chemical library against ST313 iNTS in a cell-based assay using infected RAW264.7 macrophages. Unlike our previous macrophage screen,^23^ we designed an approach to enrich for compounds with host-directed activities (Fig. 1b). Briefly, each compound in the library was added to a well of uninfected RAW264.7 macrophages for 4 h and then washed out prior to infection with opsonized ST313 iNTS engineered for luciferase expression.^27^ With slight modifications to our reported high-throughput infection method,^23^ macrophages were treated with fosfomycin following infection to prevent extracellular bacterial replication and intramacrophage bacterial viability was monitored by luminescence readings in a time series.

After normalizing each screening dataset independently and correcting for plate and well effects by interquartile-mean based methods,^28^ we directly compared the growth of ST313 over 20 h in LPM (OD_600_) to the replication (luminescence) of ST313 over 6 h in RAW264.7 macrophages after exposure to each chemical compound (Fig. 1c and Dataset S1, ESI†). We identified 92 compounds that significantly restricted ST313 growth in LPM below the mean of the dataset, and 30 compounds that similarly restricted ST313 luminescence in macrophages. Only two compounds, cetylbyridinium chloride (an antiseptic quaternary ammonium compound) and purpurogallin carboxylic acid (an oxidation product of gallic acid with anticancer activity) were active in both screens, demonstrating the selectivity of our revised macrophage screening platform to identify molecules that elicit antimicrobial activity in a host cell-dependent manner.

### Nucleoside antimetabolites inhibit ST313 iNTS under host-mimicking conditions

**Fig. 2:**
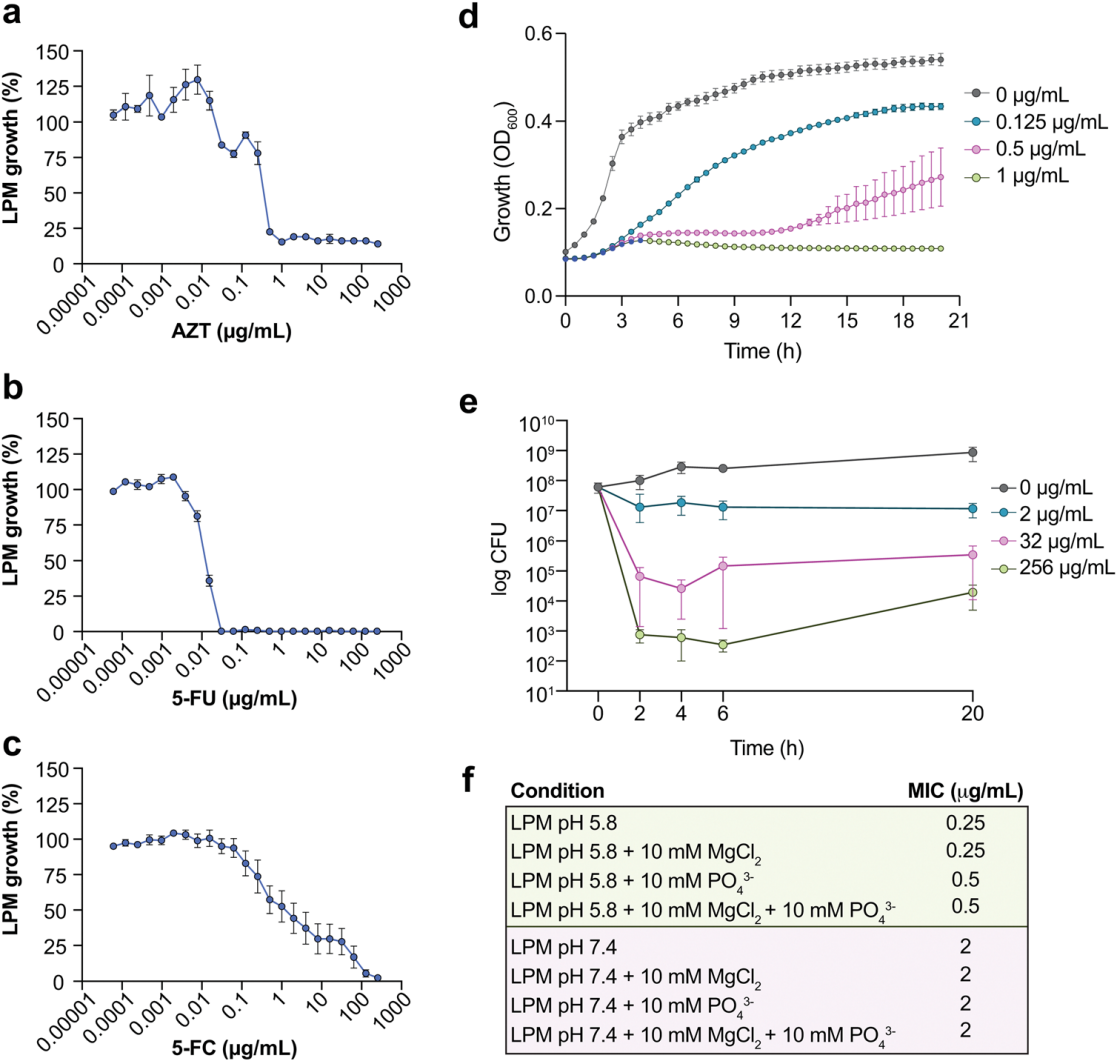
Potency analysis of AZT against ST313. (a) Potency of 3′-azido-3′-deoxythymidine (AZT) against ST313 iNTS grown in LPM. Growth is normalized to a DMSO control (set to 100%), dots and error indicate mean and s.e.m. for three biological replicates. (b) As in panel a, for 5-fluorouracil (5-FU). (c) As in panel a, for 5-fluorocytosine (5-FC). (d) Growth kinetics of ST313 iNTS in the presence of AZT. OD600 was measured at 30 min intervals for 20 h of incubation in LPM, in the presence of AZT at the concentrations indicated. Dots and error indicate mean and s.e.m. for three biological replicates. (e) Viable bacterial counts (log CFU) of ST313 iNTS measured after 0, 2, 4, 6, and 20 h of incubation in LPM in the presence of AZT at the concentrations indicated. Dots and error indicate mean and s.e.m. for three biological replicates. (f) Table of MIC values for AZT against ST313 grown in various media conditions as indicated.

The 92 compounds with antimicrobial activity against ST313 iNTS under host-mimicking conditions included quinolone antibiotics, antifungals, and other known antimicrobials. One active compound, 3′azido-3′deoxythymidine (AZT) (an azido-substituted thymidine analog and antiretroviral agent used in the treatment of HIV), was particularly potent, stable, and previously reported to have antimicrobial activity against some members of the Enterobacteriaceae family. Thus, we selected AZT as an exemplar compound to further characterize and validate our chemical screening approach. We determined that AZT inhibited ST313 growth at 0.25–0.5 μg mL^−1^ in LPM media (Fig. 2a). We tested other pyrimidine analogs including 5-fluorouracil (5-FU) and 5-fluorocytosine (5-FC) against ST313 in LPM media. Interestingly, both 5-FC and 5-FU were active against ST313 in LPM but their MICs differed by ∼3 logs (MIC_5-FU_ ∼ 0.03 μg mL^−1^, MIC_5-FC_ ∼ 100 μg mL^−1^) (Fig. 2b and c). To gain further insight into the basis of AZT activity, we monitored the growth of ST313 in the presence of AZT at sub-inhibitory concentrations during overnight incubation. This approach has been shown to generate unique dose response profiles for different antimicrobial agents, providing insight into mechanisms of bactericidal or bacteriostatic activity.^29^ In the presence of AZT at near-MIC concentrations, ST313 iNTS had an increased lag time, reduced replication, and decreased maximum optical density (Fig. 2d). Similar results were observed by assessing viable bacterial counts of ST313 on solid media after treatment with AZT at 2, 32, and 256 μg mL^−1^. Concentrations at or above 2 μg mL^−1^ reduced bacterial viability of ST313 after 6 h of incubation in a dose-dependent manner, and after 20 h of incubation there was a ∼4.5-log decrease in ST313 viability relative to untreated bacterial cells at the highest concentration tested (Fig. 2e). These findings are consistent with dose-dependent bactericidal activity of AZT against ST313. We also noted that AZT was less effective against ST313 when grown in LPM media at neutral pH or supplemented with magnesium or phosphate (Fig. 2f).

### AZT activity against ST313 iNTS synergizes with conventional antibiotics and requires bacterial thymidine kinase

**Fig. 3:**
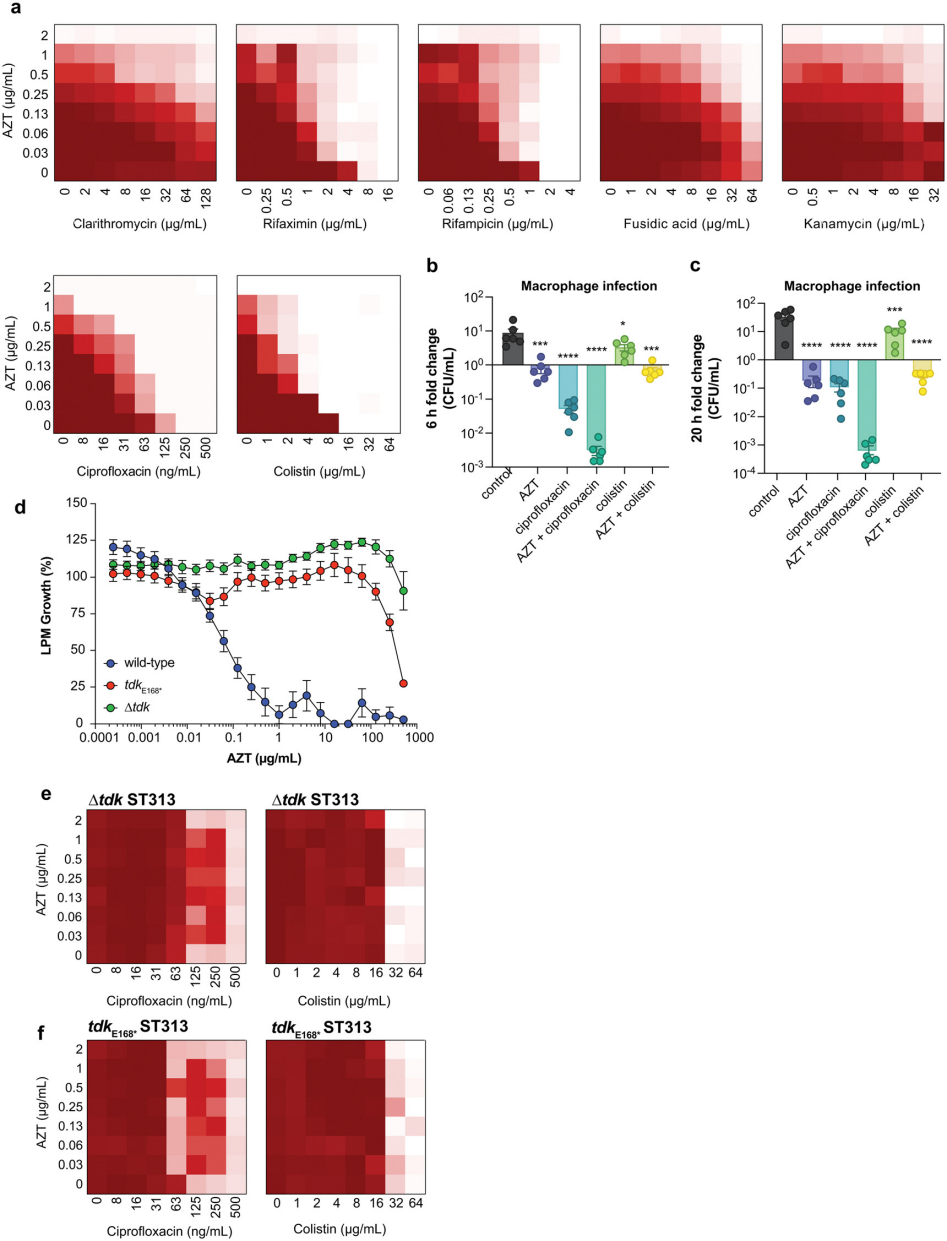
Combinatorial synergy between AZT and antibiotics is dependent on thymidine kinase activity. (a) Checkerboard broth microdilution assays between AZT and the indicated antibiotics for wild-type ST313 grown in LPM. Darker shades of red indicate higher bacterial cell density; white indicates the absence of bacterial growth. (b) Compound treatment (AZT, 128 μg mL^−1^; ciprofloxacin, 2 μg mL^−1^; colistin, 128 μg mL^−1^; individually or in combination) and bacterial infection of RAW264.7 macrophages for 6 h. Bars indicate mean and s.e.m. Groups were compared against control-treated macrophages (equivalent concentration of DMSO) *via* one-way ANOVA and corrected for multiple comparisons with Sidak's test. (c) As in panel b, for 20 h of bacterial infection. (d) Potency of AZT against wild-type, 
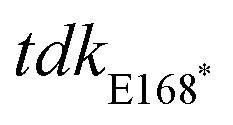
, and Δ*tdk* ST313 iNTS grown in LPM. Growth is normalized to a DMSO control (set to 100%), dots and error indicate mean and s.e.m. for three biological replicates. (e) and (f) Loss of combinatorial synergy in *tdk* mutants. Checkerboard broth microdilution assays between AZT and the indicated antibiotics for Δ*tdk* ST313 (e) and 
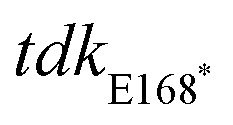
 ST313 (f) grown in LPM. Darker shades of red indicate higher bacterial cell density; white indicates the absence of bacterial growth.

Combinations of antibiotics are commonly used in medicine to broaden antimicrobial spectrum and generate synergistic effects. To better understand the potential for host-mimicking screening hits to synergize with conventional antibiotics, we tested a set of antibiotics covering several major drug classes for changes in MIC when applied in pairwise combination with AZT. Antibiotic partners included DNA-damaging molecules, macrolides, and antibiotics that inhibit transcription or translation. These experiments revealed a synergistic interaction between AZT and the DNA topoisomerase-targeting antibiotic ciprofloxacin, and with the polymyxin antibiotic colistin against ST313 (Fig. 3a). Ciprofloxacin is a fluoroquinolone antibiotic that inhibits DNA synthesis, suggesting a possible complementary effect with the nucleoside analog activity of AZT. These results are also consistent with a previous report of synergism between AZT and colistin against *E. coli* and *Klebsiella pneumoniae*.^30^ We validated this synergistic activity by testing the impact of AZT alone or in combination with ciprofloxacin or colistin against intracellular ST313. Consistent with our *in vitro* results, we found that the combination of AZT and ciprofloxacin reduced the intracellular replication of ST313 more effectively than either AZT or ciprofloxacin alone at both 6 h (Fig. 3b) and 20 h post-infection (Fig. 3c). Although the combination of AZT plus colistin was highly synergistic *in vitro*, this combination was not as effective as AZT and ciprofloxacin at reducing intracellular ST313 during infection of macrophages.

We hypothesized that the antimicrobial effect of AZT was derived from its anti-metabolite activity on nucleotide metabolism or by processing of AZT itself. To explore this hypothesis, we attempted to isolate mutants that were resistant to AZT. Serial passage of ST313 in the presence of AZT yielded one AZT-resistant colony that exhibited a shift in MIC from ∼0.5 μg mL^−1^ to >512 μg mL^−1^. Whole-genome sequencing of this strain revealed one acquired nonsense mutation (E168*) in a gene encoding for thymidine kinase (*tdk*; STMMW_17451). This finding was consistent with previous data indicating AZT and other nucleoside analogs act as prodrugs that require phosphorylation by endogenous kinases. AZT can be phosphorylated by thymidine kinase into its 5′-mono-, -di-, and -triphosphate derivatives,^31^ and only AZT-triphosphate can be incorporated into growing DNA chains to terminate chain elongation.^32^ To confirm these results, we transferred the 
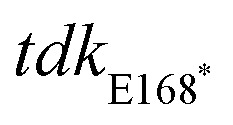
 nonsense mutation into a wild-type ST313 iNTS background by allelic replacement and also generated a Δ*tdk* mutant and tested the susceptibility of these mutant strains to AZT. Both the 
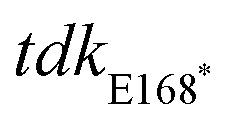
 and Δ*tdk* strains were resistant to AZT activity relative to wild-type ST313, with a >500-fold shift in MIC (Fig. 3d). These data supported the hypothesis that AZT activity against ST313 is dependent on bacterial thymidine kinase. Consistent with these findings, we observed a loss of synergy between AZT and either ciprofloxacin or colistin in both the Δ*tdk* strain (Fig. 3e) and the 
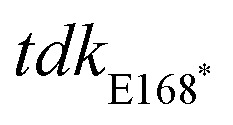
 mutant (Fig. 3f).

### Macrophage screen for host-directed actives against intracellular iNTS.

Host-informed therapies are an emerging subset of anti-infectives aimed to enhance host-dependent defense functions. To enrich for screening actives with host-directed modes of action, we pretreated macrophages with compounds, washed out the compound medium, and infected pre-treated cells with ST313 iNTS and followed intracellular replication. For these experiments, we used cultured macrophages pretreated with LPS to promote phagocytic uptake of bacteria.^33^

**Fig. 4:**
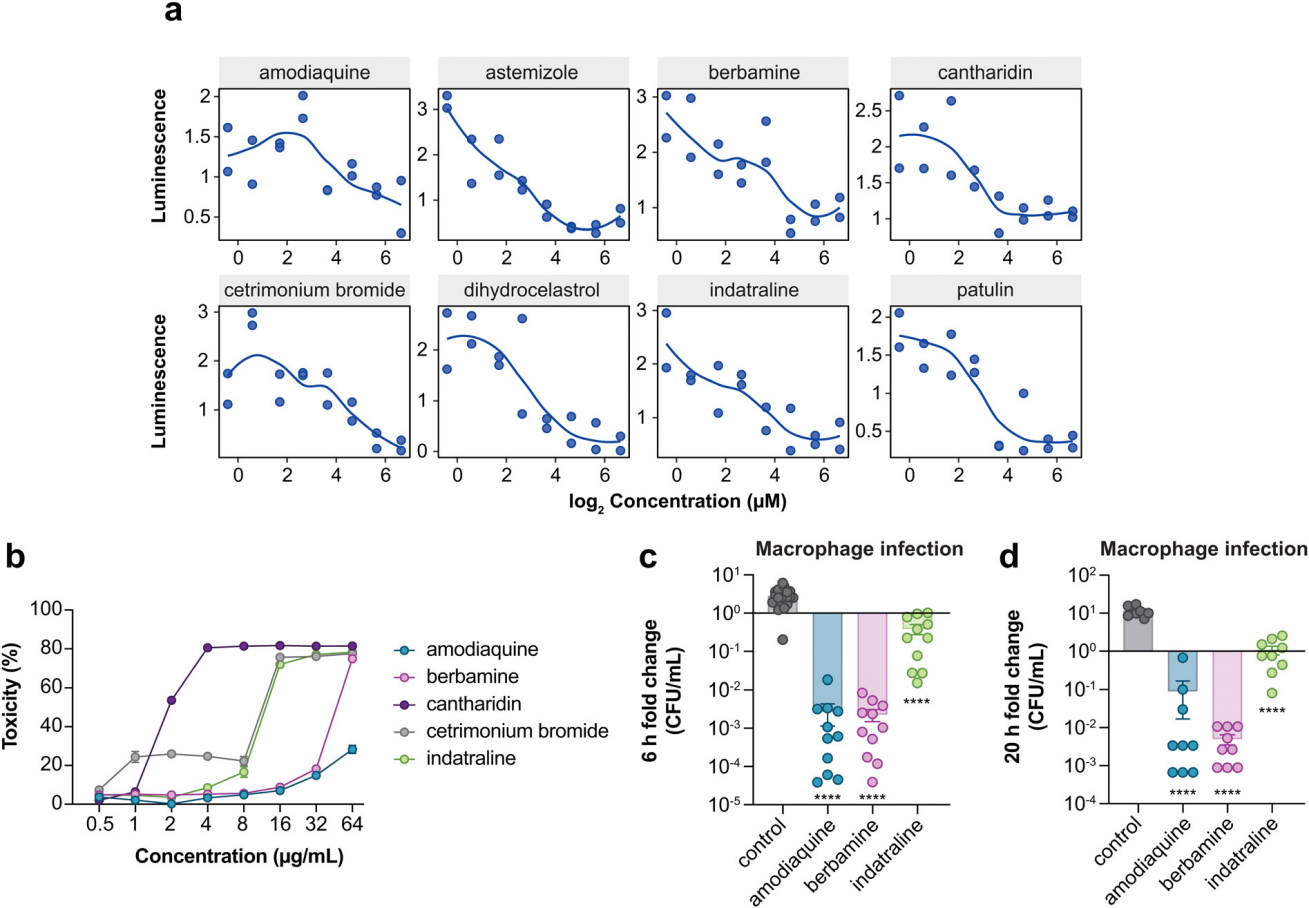
Potency analysis of macrophage-active compounds. (a) Re-screening of hit compounds against ST313 iNTS in macrophages. Dots indicate averaged bacterial luminescence from two technical replicates. (b) Percentage cytotoxicity in RAW264.7 macrophages after exposure to indicated compounds. Dots indicate mean and s.e.m. from three independent experiments. (c) Compound treatment and bacterial infection of RAW264.7 macrophages for 6 h. Bars indicate mean and s.e.m. Groups were compared against control-treated macrophages (equivalent concentration of DMSO) *via* one-way ANOVA and corrected for multiple comparisons with Sidak's test. ****p < 0.0001. (d) As in panel c, for 20 h of bacterial infection.

This screen identified 28 compounds that significantly restricted intracellular replication of ST313 in macrophages, none of which were hits in the LPM medium screens (Fig. 1c). Given how closely LPM approximates the chemical conditions of the SCV, we reasoned that compounds with activity in macrophages but not LPM are likely to have host-targeting modes of activity. To further investigate these putative actives, we re-ordered and re-screened all 28 compounds for potency and found that 8 compounds resulted in a dose-dependent reduction in intramacrophage ST313 luminescence over a 6 h period (Fig. 4a). Based on known activity, chemical diversity, and commercial availability, we selected five compounds for further characterization (amodiaquine, berbamine, cantharidin, cetrimonium bromide, indatraline). We determined the cytotoxicity of each compound towards macrophages using lactate dehydrogenase (LDH) release, as host cell toxicity could impact bacterial luminescence in a manner unrelated to the host pathways of interest. From these data, we excluded cantharidin and cetrimonium bromide from further experimentation because both compounds resulted in >20% cytotoxicity at or below 4 μg mL^−1^ (Fig. 4b). We also identified a maximum, non-toxic concentration for the remaining three compounds to be used in secondary assays (32 μg mL^−1^ for amodiaquine and berbamine, and 8 μg mL^−1^ for indatraline).

We next validated the ability of the remaining three compounds to attenuate intracellular ST313 replication by enumerating bacterial viability directly. Following a similar infection protocol used in our high-throughput macrophage screen, we pre-treated macrophages separately with the three priority compounds at the maximum concentrations described above, then removed each compound and infected macrophages with ST313 iNTS to enumerate viable intracellular bacteria at 0, 6, and 20 h post-infection. Consistent with our luminescence screening results, pre-treatment of macrophages with each compound significantly reduced the intracellular replication of ST313 at 6 and 20 h post-infection (Fig. 4c and d).

### Specificity and immunomodulatory activity of host-directed compounds

**Fig. 5:**
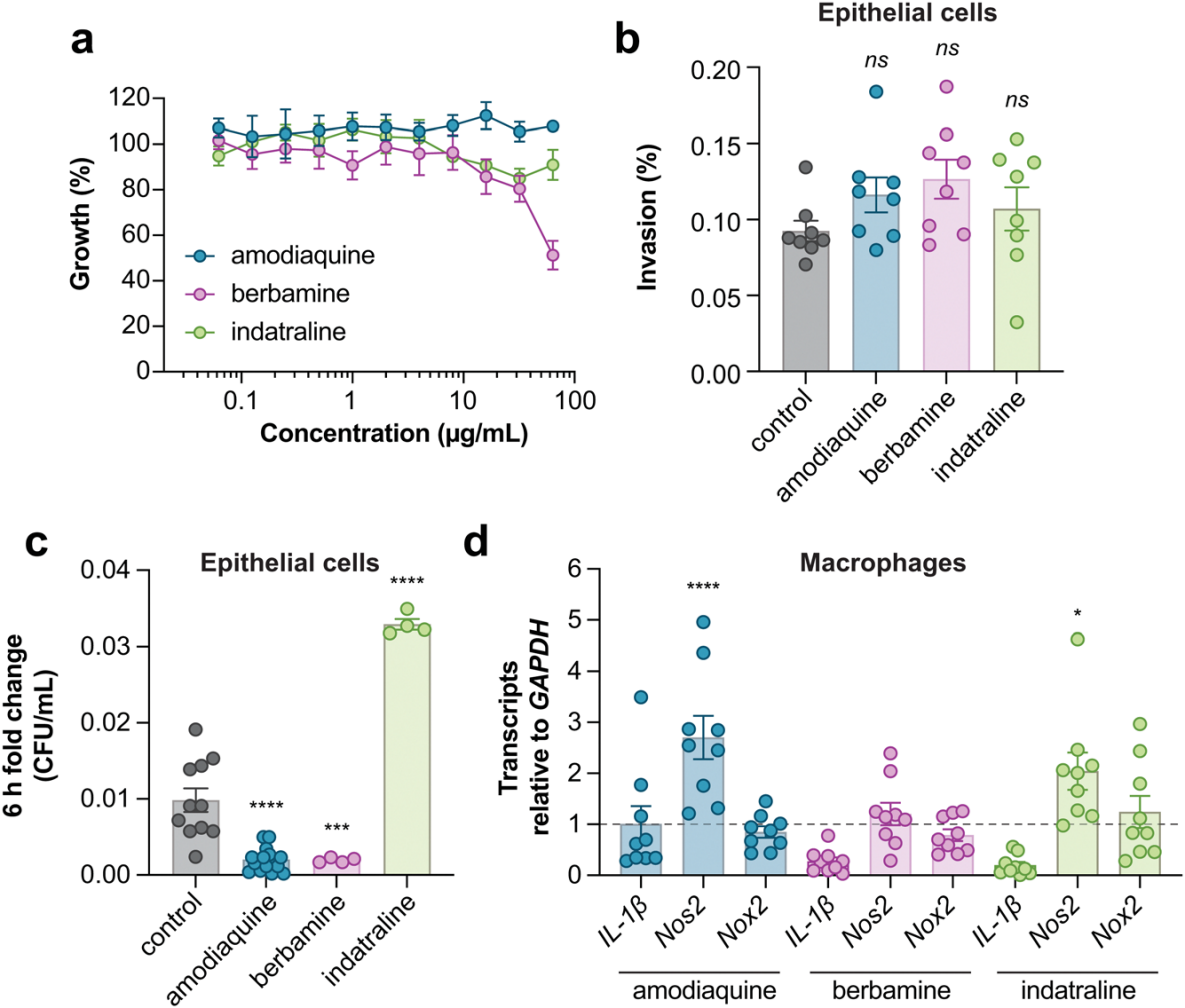
Specificity and immunomodulatory activity of macrophage-active compounds. (a) Growth normalized to a DMSO control (set to 100%) of ST313 in the presence of indicated compounds. Data is from four biological replicates, dots and error indicate mean and s.e.m. (b) Compound treatment and bacterial infection of HeLa cells. Bars indicate mean and s.e.m. of invasion as a percentage. Groups were compared against control-treated macrophages (equivalent concentration of DMSO) *via* one-way ANOVA and corrected for multiple comparisons with Dunnett's test. (c) As in panel b, bars indicate mean and s.e.m. of CFU per mL reported as a fold change over 6 h of replication. ***p < 0.001, ****p < 0.0001. (d) Gene expression measured by RT-qPCR. Bars indicate mean and s.e.m. Groups were compared against a value of 1 (control-treated macrophages) *via* one-way ANOVA and corrected for multiple comparisons with Dunnett's test. *p < 0.05, ****p < 0.0001.

The macrophage screen was designed to enrich for host-directed active molecules and the low overlap of hits between this screen and the host-mimicking media screen is consistent with having achieved this. However, it remained possible that some active compounds may accumulate within macrophages and directly target bacterial viability in a manner that still requires host-dependent pathways. To examine the specificity of amodiaquine, berbamine, and indatraline against intramacrophage iNTS, we tested these compounds against extracellular iNTS grown in tissue culture media. A result where a compound was inactive in the same experimental media in the absence of host cells would provide more support for a host-directed mode of action. At the highest compound concentration tested, only berbamine dosed in tissue culture medium inhibited bacterial growth to ∼50% relative to untreated bacteria, while amodiaquine and indatraline resulted in ∼0 and 20% growth inhibition, respectively (Fig. 5a). These data are consistent with the inhibitory activity of these compounds against intracellular iNTS, particularly for amodiaquine and indatraline, being directed through macrophage-dependent activities.

To examine the specificity of macrophages to potentiate the antibacterial activities of amodiaquine, berbamine, and indatraline, we repeated these experiments with epithelial cells. We considered that any difference in compound activity between macrophages and epithelial cells might suggest that the compound targeted a macrophage-specific process, such as phagocytosis or another innate host defense function. In these experiments, none of the compounds inhibited bacterial invasion into epithelial cells (Fig. 5b), however, like infected macrophages, bacterial replication was significantly inhibited in epithelial cells treated with amodiaquine or berbamine. We found that epithelial cells treated with indatraline supported better ST313 iNTS growth as compared to untreated cells, which was unexpected (Fig. 5c). Together, these data suggest that amodiaquine and berbamine may modulate a host defense pathway that is conserved between epithelial cells and macrophages, while indatraline may impact either a distinct pathway(s) in these cell types or the same pathway that functions in different ways to control intracellular bacterial growth.

Each of the three host-dependent compounds have known physiological activities separate from their antibacterial effect revealed here: amodiaquine is an anti-malarial drug, berbamine is an anti-cancer drug, and indatraline is an anti-depressant. To explore other host-based targets of these active compounds—especially the possible impact of each compound on macrophage defense functions—we measured the transcription of three key genes in RAW264.7 macrophages that are involved in innate host defenses: the pro-inflammatory cytokine IL-1β, inducible nitric oxide synthase (Nos2), and NADPH oxidase (Nox2). In these experiments, we observed that both amodiaquine and indatraline significantly increased Nos2 expression (Fig. 5d). More work remains to be done to fully characterize the mechanisms of action of these compounds. However, the increased expression of this inflammatory response gene suggests that nitric oxide-dependent host defenses may play a role in clearing the intracellular compartment of invasive bacteria following administration of amodiaquine or indatraline. These preliminary results provide possible mechanistic routes to pursue in future experimental work.

## Discussion

Bloodstream infections caused by iNTS *Salmonella* are a major cause for concern. Due to the high prevalence of multidrug resistance in this *Salmonella* pathovariant, developing new therapeutics against iNTS requires an exploration of unconventional screening approaches and a deeper understanding of chemical sensitivity under infection-relevant growth conditions. In this work, we developed and implemented small molecule screens in both host-mimicking media and in cultured macrophages to explore the chemical sensitivity of iNTS ST313 under conditions that are physiologically relevant to the host.

We identified 92 compounds that possessed antimicrobial activity against ST313 grown in LPM, illustrating a possible strategy to overcome multidrug resistance in iNTS. To validate this approach, we characterized the antiretroviral nucleoside analog AZT as a potent exemplary active from this screen. We found that AZT restricted ST313 replication in a thymidine kinase-dependent manner. We also discovered three compounds, amodiaquine, berbamine, and indatraline, that significantly restricted the intracellular replication of ST313 in cultured macrophages with activity consistent with a host-directed mode of action. Our results indicated that one or more of these compounds might help bolster host immune processes, however more work is required to establish a mechanistic understanding of intracellular bacteria growth restriction, and to interrogate the balance between possible host- and bacterial-targeting modes of activity for host-directed compounds. Preliminary evidence suggests at least two of these compounds may augment nitric oxide-dependent macrophage defense functions to restrict intracellular *Salmonella*.

The AZT hit from the host-mimicking media screen provided a proof-of-concept molecule for follow up due to prior literature in other Gram-negative pathogens, including *Escherichia*, *Klebsiella*, *Shigella*, *Enterobacter*, and even non-multidrug resistant strains of *Salmonella*. Previous studies have examined AZT activity in macrophages and murine infection models with other types of *Salmonella*. For example, AZT was shown to reduce intracellular replication of *Salmonella* (of an unknown sequence type) in cultured macrophages after 24 h of incubation,^34^ and subcutaneous administration of AZT reduced *Salmonella* burden in infected calves.^35^ These data, combined with our results, suggests that nucleoside anti-metabolism might be a promising lead against ST313 iNTS, especially in the context of a combination therapy strategy. Indeed, given the synergistic activity we revealed between AZT and colistin or ciprofloxacin *in vitro*, a deeper exploration of combination therapy for ST313 is warranted.

Differences in bacterial sensitivity to nucleoside analogs have been linked to variation in the number and substrate specificities of endogenous nucleoside kinases.^31,36^ Indeed, our suppressor experiments yielded an AZT-resistant mutation in the *tdk* thymidine kinase, and this was confirmed with additional mutational analysis. A previous transcriptomics study identified expression differences in metabolic genes between ST313 and ST19, including the downregulation of genes involved in uracil and cytosine uptake (*uraB, codB*), melibiose utilization (*melAB*), carbamoyl-phosphate metabolism and pyrimidine biosynthesis (*carAB, pyrEIB*), nitrate reductase (*napDF*), and sulfate metabolism (*cysPU* and *sbp*).^12^ ST313 was also previously shown to grow less efficiently than ST19 using purine and pyrimidine as phosphorus sources.^37^ These studies, in conjunction with our results here, indicate that unique transcriptional profiles of ST313 may produce phenotypes that are susceptible to chemical inhibition. Future experiments directed at understanding the regulation and activity of metabolic and other pathways in iNTS could reveal new entry points into the discovery of anti-ST313 compounds.

Our second chemical screen performed in cultured macrophages was designed to enrich for compounds with host-directed activities. Considering the systemic nature of ST313 iNTS, we reasoned that compounds that interacted with macrophage defense responses to bacterial infection may be particularly promising leads. We identified three compounds (amodiaquine, berbamine, and indatraline) that restricted intracellular replication of ST313. Our preliminary results suggest that these compounds may exert immunomodulatory effects on host cells to increase their innate defense functions, indirectly restricting the growth of ST313 iNTS. However, more work is required to better understand the mechanism(s) of action. The known physiological targets of these compounds bolsters this speculation, indicating a likelihood of interaction with immune targets: amodiaquine is an aminoquinoline derivative with anti-malarial and anti-inflammatory properties,^38^ berbamine is an anti-cancer drug with inhibitory activity towards a *bcr*/*abl* fusion gene, NF-κB, and IL-1,^39–41^ and indatraline is a non-selective monoamine transporter that blocks dopamine, norepinephrine, and serotonin reuptake^42^ and induces autophagy.^43^ Understanding the full extent of the host processes affected by these compounds requires further experimentation, including transcriptional profiling of macrophages and other cell types. Future work should also be directed to investigating the kinetics of compound activity when administered at different stages of infection. It remains possible, for example, that host-directed compounds may possess different modes of action if administered at later of infection. Moreover, further experimentation is required to determine whether active compounds reprogram macrophages and/or epithelial cells to possess either bacteriostatic or bactericidal activity against intracellular ST313.

Host-directed therapeutics comprise chemical agents that either enhance protective host functions or blunt inflammatory processes that cause damage.^44^ Apart from studies focusing on host-directed therapies as treatments for tuberculosis^44^ and pneumococcal pneumonia,^45^ host-directed therapies have been relatively underexplored adjuncts to anti-infective therapy. The success and promise of host-directed therapies to modulate innate immune functions in other therapeutic areas including transplantation, autoimmune diseases, and cancer^46,47^ warrants a critical exploration of their potential against drug-resistant infections such as those caused by iNTS. The work presented here provides a high-throughput platform and screening approach to probe even greater chemical space towards this goal.

## Experimental

### Bacterial strains and culture conditions

*Salmonella* experiments were performed with strain D23580 (ST313). For compound screening in macrophages and secondary assays, this strain was transformed with pGEN-*lux* conferring gentamicin resistance.^27^ Routine propagation of bacteria was in LB media (10 g L^−1^ NaCl, 10 g L^−1^ Tryptone, 5 g L^−1^ yeast extract) supplemented with appropriate antibiotics (streptomycin, 100 μg mL^−1^, gentamicin, 15 μg mL^−1^). Where indicated, bacteria were grown in LPM^25^ (acidic pH, low phosphate, low Mg^2+^ media) (5 mM KCl, 7.5 mM (NH_4_)_2_SO_4_, 0.5 mM K_2_SO_4_, 80 mM MES pH 5.8, 0.1% casamino acids, 0.3% (v/v) glycerol, 24 μM MgCl_2_, 337 μM PO_4_^3−^). Bacteria were grown at 37 °C.

### Cell culture maintenance

Cells were maintained in a humidified incubator at 37 °C with 5% CO_2_. HeLa epithelial cells and RAW264.7 macrophages were grown in DMEM containing 10% FBS (Gibco) and seeded in tissue culture-treated 96-well (100 μL per well, 10^5^ cells per well) or 384-well (50 μL per well, 5 × 10^4^ cells per well) plates (Corning) ∼20–24 h prior to use. In experiments with RAW264.7 macrophages, cells were pretreated with 100 ng mL^−1^ LPS from *Salmonella enterica* serovar Minnesota R595 (Millipore) for ∼20–24 h prior to infection.

### Screening reagents

All high-throughput compound screening was performed at the Centre for Microbial Chemical Biology (McMaster University). The chemical library we screened contained 3840 diverse small molecules assembled from Sigma-Aldrich and MicroSource. Screening stocks (5 mM) were stored at −20 °C in DMSO. The following compounds were ordered from Sigma-Aldrich: 3′-azido-3′-deoxythymidine (AZT), 5-fluorouracil (5-FU), 5-flurocytosine (5-FC), ciprofloxacin, colistin, amodiaquine, cantharidin, indatraline. Berbamine and cetrimonium bromide were sourced from Cedarlane. Compounds were routinely dissolved in DMSO at a concentration of 10 mg mL^−1^ and stored at −20 °C.

### LPM chemical screening

Overnight cultures of ST313 iNTS were sub-cultured ∼1 : 50 in LB and grown for 2–2.5 h. The cultures were diluted ∼1 : 350 into LPM and dispensed into 384-well black, clear flat bottom plates (Corning) to a final volume of 30 μL per well. Sixty nL of each compound (5 mM stocks) was added using an Echo 550 Liquid Handler directly into wells for a final concentration of 10 μM compound per well. OD_600_ was read immediately after compound addition (OD_0h_) and after ∼20 h of incubation at 37 °C (OD_20h_). Normalized growth was calculated by subtracting OD_0h_ from OD_20h_, then correcting for plate and well effects by interquartile-mean based methods.^28^ Compounds that reduced growth more than 2.65 s.d. below the mean of the dataset were considered actives. Screening was performed in duplicate.

### MIC determination for nucleoside analogs

ST313 cultures were grown overnight in LB, then diluted ∼1 : 10 000 into LPM or LB. AZT, 5-FU, and 5-FC were serially diluted two-fold starting at 128 μg mL^−1^ to a final concentration of <0.0001 μg mL^−1^, then added to bacteria-containing media. OD_600_ was read immediately after compound addition (OD_0h_) and after ∼20 h of incubation at 37 °C (OD_20h_). Percentage growth was calculated by subtracting OD_0h_ from OD_20h_, then normalizing values to a DMSO control set to 100% growth.

### Monitoring kinetics of AZT-induced bacterial death

ST313 cultures were grown overnight in LB, then sub-cultured into LPM. AZT was added to bacteria-containing media at 1, 0.5, and 0.125 μg mL^−1^. OD_600_ was read immediately after compound addition, then every 30 min for 20 h of incubation at 37 °C while shaking. At 0, 2, 4, 6, and 20 h, cultures were serially diluted and plated on LB agar to enumerate viable bacterial CFU.

### Checkerboard broth microdilution assays

8 × 8 matrices of compound were created in 96-well plates (Corning) with two-fold serial dilutions of AZT and various partner antibiotics at 8 concentrations. After overnight growth in LB, bacteria were diluted ∼1 : 5000 into LPM or LB and added to each well of the 8 × 8 matrix. After addition of bacteria, plates were incubated at 37 °C for ∼20–22 h, before and after which OD_600_ was measured. At least two biological replicates were performed for each assay.

### Macrophage experiments with AZT, ciprofloxacin, and colistin

Cells were maintained in a humidified incubator at 37 °C with 5% CO_2_. RAW264.7 macrophages were grown in DMEM containing 10% FBS. At 20 hours prior to infection, cells were seeded at 10^5^ cells per well in tissue culture-treated 96-well plates. Media was supplemented with 100 ng mL^−1^ LPS from *Salmonella enterica* serovar Minnesota R595. Cultures of ST313 were grown overnight in LB, pelleted, washed once with phosphate-buffered saline (PBS), and resuspended in PBS. Bacteria were opsonized in 20% normal human serum in PBS for 30 minutes at 37 °C. 100 μL of opsonized bacteria (diluted in DMEM + 10% FBS to achieve a multiplicity of infection (MOI) of 50 : 1) was added to each well of RAW264.7 macrophages. Plates were centrifuged at 200 × *g* for 2 minutes, then incubated for 30 minutes at 37 °C with 5% CO_2_. Bacteria-containing media was then removed from macrophages and replaced with 100 μL per well of DMEM + 10% FBS + 100 μg mL^−1^ fosfomycin to eliminate extracellular bacteria. Plates were incubated again for 30 minutes at 37 °C with 5% CO_2_. For 0 hour samples, fosfomycin-containing media was removed, cells were gently washed twice with PBS, and lysed in sterile MilliQ water. Bacterial CFU from each lysed well were enumerated by serially diluting in PBS and plating on LB agar with appropriate selection. Fosfomycin-containing media was removed from the remaining wells and replaced with 100 μL per well of DMEM + 10% FBS + 10 μg mL^−1^ fosfomycin + AZT at 128 μg mL^−1^, ciprofloxacin at 2 μg mL^−1^, colistin at 128 μg mL^−1^, AZT at 128 μg mL^−1^ and ciprofloxacin at 2 μg mL^−1^, or AZT at 128 μg mL^−1^ and colistin at 128 μg mL^−1^. After 6 hours of incubation at 37 °C with 5% CO_2_, remaining wells were washed twice with PBS, then lysed in sterile MilliQ water for plating and CFU enumeration. For experiments with 20 hours of incubation, an identical protocol was followed, except for a modified MOI of 20 : 1 to prevent excessive macrophage lysis overnight. Each sample was done in technical replicates across three biological replicates.

### Suppressor isolation and whole-genome sequencing

Spontaneous resistant mutants to AZT were selected for by serial passage in liquid culture. An ST313 culture was grown overnight in LPM at 37 °C, then diluted ∼1 : 2000 into 200 μL LPM per well in 96-well plates (Corning) containing two-fold serial dilutions of AZT beginning at 256 μg mL^−1^, and incubated overnight at 37 °C. The susceptibility of this strain to AZT was repeatedly tested for several days: every other day, the well with observable growth at the highest concentration of AZT was sub-cultured and grown overnight at 37 °C in LPM containing the corresponding AZT concentration at which growth was observed. When the strain displayed resistance to >256 μg mL^−1^ AZT, genomic DNA was extracted using the QIAamp DNA mini kit (Qiagen). Samples were sequenced on a MiSeq 2 × 250 platform with paired-end reads. Raw reads were processed with FastQC and trimmed with Cutadapt^48^ to remove Nextera transposase sequences. Sequencing data was aligned against the reference genome for *Salmonella* ST313 (FN424405) and analyzed using breseq^49^ in polymorphism mode with default settings.

### Cloning and mutant generation

All DNA manipulation procedures followed standard molecular biology protocols. Primers were synthesized by Sigma-Aldrich. PCR was performed with Phusion, Phire II, or Taq DNA polymerases (ThermoFisher). All deletions were confirmed by PCR and verified by DNA sequencing performed by Genewiz Incorporated. An unmarked, in-frame gene deletion mutant of the *tdk* thymidine kinase gene (STMMW_17451) was generated *via* homologous recombination from a suicide plasmid as described previously.^3^ Briefly, ∼500 bp upstream and downstream of the target gene were PCR-amplified and spliced together by stand overlap-extension PCR, using the following primers: Δ*tdk* upstream F (5′-GGGGAGCTCAGCTGGGTATTCCTAAGTCTATCC-3′), Δ*tdk* upstream R (5′-TACCTGAGGTAAAGAGCGGCTTAT-3′), Δ*tdk* downstream F (5′-TTGGTCGCAGGACCTCACCTGA-3′), Δ*tdk* downstream R (5′-gggGGTACCGGGGGATACTCACCGTCTGTCGCT-3′). The resulting deletion allele was digested with KpnI/SacI, ligated into KpnI/SacI digested suicide plasmid pRE112 (Edwards *et al.*, 1998), and recovered in *E. coli* DH5α λ pir. The sequence-verified construct was transformed into *E. coli* SM10λ *pir* to create a donor strain for conjugation and introduced into wild-type ST313 iNTS *via* conjugal transfer. Merodiploid clones were first selected on streptomycin and gentamicin, followed by selection for mutants using SacB-based counterselection on 10% (w/v) sucrose and growth at 30 °C.^50^ Similar methods were used for generation of the 
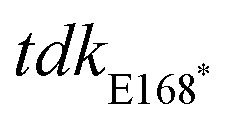
 mutant, introducing the point mutation by overlap-extension PCR and chromosomal replacement by allelic exchange, using the following primers: 
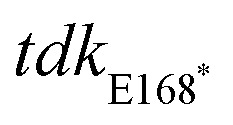
 upstream F (5′-GGGGAGCTCCTGTCTGAAGATGCCTTCGATGAC-3′), 
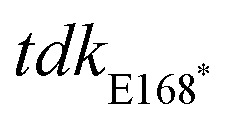
 upstream R (5′-CCTTGATCAGGACGGCAGGCCTTATAACAGAGGCGAACAGGTGGT-3′), 
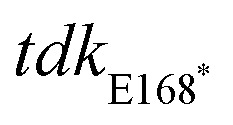
 downstream F (5′-GTTCATTCCCGCCAATAACCACCTGTTCGCCTCTGTTATAAGGC-3′), 
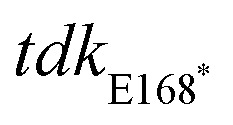
 downstream R (5′-GGGGGTACCCAATGAATGCGGGTAAGTCGACTGC-3′).

### Macrophage chemical screening

100 nL of each compound (5 mM stocks) was added using an Echo 550 Liquid Handler directly to 384-well black, clear flat-bottom plates (Corning) containing LPS-pretreated RAW264.7 macrophages in 50 μL DMEM + 10% FBS, for a final concentration of 10 μM compound per well. Macrophages were incubated with compounds for 4 h at 37 °C with 5% CO_2_. Cultures of ST313 expressing pGEN-*lux* were grown overnight in LB with 15 μg mL^−1^ gentamicin. Thirty min prior to infection (∼3.5 h after compound addition), bacteria were opsonized for 30 min in 20% human serum (Innovative Research) in PBS at 37 °C. Compound-containing media was then removed from macrophages and replaced with 50 μL per well of opsonized bacteria (diluted in DMEM + 10% FBS to achieve an MOI of 50 : 1). Plates were centrifuged at 200 × *g* for 3 min, then incubated for 30 min at 37 °C with 5% CO_2_. Bacteria-containing media was removed from macrophages and replaced with 50 μL per well of DMEM + 10% FBS + 100 μg mL^−1^ fosfomycin to eliminate extracellular bacteria. Plates were incubated again for 30 min at 37 °C with 5% CO_2_. Fosfomycin-containing media was removed from macrophages and replaced with 50 μL per well of DMEM + 10% FBS + 10 μg mL^−1^ fosfomycin. Luminescence was read immediately after this media replacement step (Lux_0h_), plates were incubated for 6 h at 37 °C with 5% CO_2_, then luminescence was measured a second time after 6 h (Lux_6h_). Normalized luminescence was calculated by dividing Lux_6h_ by Lux_0h_ (to represent fold change increase over the course of the experiment), then correcting for plate and well effects by interquartile-mean based methods.^28^ Compounds that reduced growth to 50% or less than the mean of the dataset were considered actives. Screening was performed in duplicate. For secondary screening of hit compounds, an identical protocol to the initial macrophage screen was followed, with the exception that compounds were serially diluted two-fold starting at 100 μM to a final concentration of 0.78 μM in DMEM + 10% FBS prior to the 4 h incubation period with macrophages.

### Cytotoxicity assays

LPS-pretreated RAW264.7 macrophages were seeded into 96-well plates in DMEM + 10% FBS as described above. Compounds were serially diluted two-fold starting at 64 μg mL^−1^ to a final concentration of 0.5 μg mL^−1^, then added directly to macrophages. After 4 h of incubation with compounds at 37 °C with 5% CO_2_, plates were centrifuged at 500 × *g* for 2 min and culture supernatant was collected for quantification of lactate dehydrogenase (LDH) release. Cytotoxicity was quantified using a colorimetric assay (G-biosciences Cytoscan^TM^-LDH Cytotoxicity Assay) wherein LDH activity is measured by recording A_490_ after 20 min incubation with substrate mix at 37 °C. Lysis control wells were treated with 10X lysis buffer for 45 min prior to supernatant collection. Percent cytotoxicity was calculated with the formula:
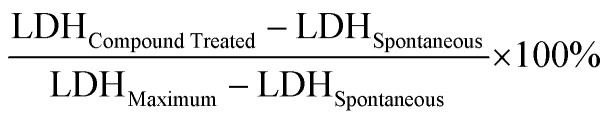
where LDH_Spontaneous_ is the amount of LDH activity in the supernatant of untreated cells and LDH_Maximum_ is the amount of LDH activity in the supernatant of lysis control wells. The LDH activity in cell-free culture medium was subtracted from each value prior to normalization to account for any serum effects.

### Intramacrophage bacterial enumeration

LPS-pretreated RAW264.7 macrophages were seeded into 96-well plates in DMEM + 10% FBS as described above. A single concentration of each compound (determined based on cytotoxicity testing) was added directly to wells, with an equivalent volume of DMSO added to control wells. Macrophages were incubated with compounds for 4 h at 37 °C with 5% CO_2_. Cultures of ST313 were grown overnight in LB, then 30 min prior to infection (∼3.5 h after compound addition), bacteria were opsonized for 30 min in 20% human serum (Innovative Research) in PBS at 37 °C. Compound-containing media was then removed from macrophages and replaced with 100 μL per well of opsonized bacteria (diluted in DMEM + 10% FBS to achieve an MOI of 50 : 1). Plates were centrifuged at 200 × *g* for 3 min, then incubated for 30 min at 37 °C with 5% CO_2_. Bacteria-containing media was removed from macrophages and replaced with 100 μL per well of DMEM + 10% FBS + 100 μg mL^−1^ fosfomycin to eliminate extracellular bacteria. Plates were incubated again for 30 min at 37 °C with 5% CO_2_. Fosfomycin-containing media was removed from macrophages and replaced with 100 μL per well of DMEM + 10% FBS + 10 μg mL^−1^ fosfomycin. Immediately after this media replacement step, adhered macrophages from half of the wells were lysed in sterile water. Bacterial CFU from each lysed well were enumerated by serially diluting in PBS and plating on LB agar (CFU at 0 h). After 6 h of incubation at 37 °C with 5% CO_2_, adhered macrophages from the other half of the wells were lysed in sterile water for plating and CFU enumeration. Fold change in CFU per mL was calculated (CFU at 6 h divided by at 0 h) to represent replication over the course of the experiment. For experiments with 20 h of incubation, an identical protocol was followed, except for a modified MOI of 20 : 1 to prevent excessive macrophage lysis.

### MIC determination for amodiaquine, berbamine, indatraline

A culture of ST313 was grown overnight in LB, then diluted ∼1 : 10 000 into DMEM + 10% FBS. Compounds were serially diluted two-fold starting at 64 μg mL^−1^ to a final concentration of <0.1 μg mL^−1^, then added to bacteria-containing media. OD_600_ was read immediately after compound addition (OD_0h_) and after ∼20 h of incubation at 37 °C (OD_20h_). Percentage growth was calculated by subtracting OD_0h_ from OD_20h_, then normalizing values to a DMSO control (set to 100%).

### HeLa epithelial cell infections and compound treatment

HeLa epithelial cells were seeded into 96-well plates in DMEM + 10% FBS as described above. A single concentration of each compound (determined based on cytotoxicity testing) was added directly to wells, with an equivalent volume of DMSO added to control wells. HeLa cells were incubated with compounds for 4 h at 37 °C with 5% CO_2_. A culture of ST313 was grown overnight in LB, then sub-cultured ∼1 : 50 for ∼2.5 h (beginning ∼1.5 h after compound addition) at 37 °C. Compound-containing media was then removed from HeLa cells and replaced with 100 μL per well of bacteria (diluted in DMEM + 10% FBS to achieve an MOI of 100 : 1). Plates were centrifuged at 500 × *g* for 2 min, then incubated for 10 min at 37 °C with 5% CO_2_. Bacteria diluted to the appropriate MOI were serially diluted in PBS and plated on LB agar to enumerate CFU (CFU_input_). Bacteria-containing media was then removed, plates were washed 3 times with PBS, then media was replaced with 100 μL per well of DMEM + 10% FBS and plates were incubated for 20 min at 37 °C with 5% CO_2_. Media was then removed and replaced with 100 μL per well of DMEM + 10% FBS + 100 μg mL^−1^ fosfomycin to eliminate extracellular bacteria. Plates were incubated again for 30 min at 37 °C with 5% CO_2_. Fosfomycin-containing media was removed, plates were washed once with PBS, and the media was replaced with 100 μL per well of DMEM + 10% FBS + 10 μg mL^−1^ fosfomycin. Immediately after this media replacement step, adhered HeLa cells from half of the wells were lysed in PBS containing 1% (v/v) Triton-X100 and 0.1% (w/v) SDS. Bacterial CFU from each lysed well were enumerated by serially diluting in PBS and plating on LB agar (CFU_0h_). After 6 h of incubation at 37 °C with 5% CO_2_, adhered HeLa cells from the other half of the wells were lysed in PBS containing 1% (v/v) Triton-X100 and 0.1% (w/v) SDS for plating and CFU enumeration (CFU_6h_). Percent invasion was quantified by dividing CFU_0h_ by CFU_input_; fold change in CFU per mL was calculated by dividing CFU_6h_ by CFU_0h_.

### RNA isolation and RT-qPCR

LPS-pretreated RAW264.7 macrophages were seeded into 96-well plates in DMEM + 10% FBS as described above. A single concentration of each compound (determined based on cytotoxicity testing) was added directly to wells with 3 technical replicates, with an equivalent volume of DMSO added to control wells. Macrophages were incubated with compounds for 4 h at 37 °C with 5% CO_2_. Compound-containing media was removed, then adhered macrophages were scraped and resuspended in 100 μL Trizol (Invitrogen) for cell lysis. RNA was extracted by chloroform separation, precipitated with 100% isopropanol and washed with 75% ethanol before treatment with DNase I (Turbo DNA-free kit). DNase I was inactivated with 2.5 mM EDTA and RNA was resuspended in DEPC water. For RT-qPCR experiments, cDNA was synthesized from purified RNA using qScript cDNA Supermix (Quantabio) and diluted 1 : 10 before use. *GAPDH* was used for normalization, RT-qPCR was performed in a LightCycler 480 (Roche) with PerfeCTa SYBR Green Supermix (Quantabio). For all experiments, normalized ratios (compound/DMSO) were calculated relative to *GAPDH* transcript levels.

### Quantification and statistical analysis

Data were analyzed using RStudio version 1.0.143 with R version 3.2.2, and GraphPad Prism 8.0 software (GraphPad Inc., San Diego, CA). Each figure legend contains information on the type of statistical test used as well as mean and dispersion measures. *P* values of <0.05 were considered significant.

## Conclusions

Overall, our findings add to a growing body of work characterizing the fitness of ST313 iNTS under host-relevant conditions and revealed novel chemical sensitivity in iNTS when screened using unconventional approaches. Our data show that infection-relevant growth conditions expose bacterial vulnerabilities in iNTS that can be exploited by small molecule targeting, whether through direct antimicrobial action or by targeting host pathways. Larger chemical libraries used in conjunction with the screening approaches described here will allow for a more comprehensive chemical susceptibility profile of ST313 iNTS. We consider these results to be encouraging for future therapeutic development to overcome existing and widespread multidrug resistance in iNTS and to accelerate urgent exploration of new targets to combat iNTS disease.

## Author contributions

C. N. T.: conceptualization, methodology, formal analysis, investigation, writing (original draft and editing), visualization; M.-A. M: validation, investigation, writing (review and editing); C. R. M.: resources; J. N. P.: investigation; E. D. B.: resources, methodology, supervision; B. K. C.: conceptualization, methodology, writing (review and editing), visualization, supervision, project administration, funding acquisition.

## Conflicts of interest

The authors have no conflicts to declare.

## Supplementary Material

CB-004-D3CB00014A-s001
